# A deep learning-based model for interval prediction of real-time clearing price in electricity market

**DOI:** 10.1016/j.isci.2026.117075

**Published:** 2026-08-10

**Authors:** Jun Yang, Jian Zhou, Lei Zhu

**Affiliations:** 1 National College for Excellent Engineers, Beihang University, Beijing 100191, China; 2 School of Management Engineering, Qingdao University of Technology, Qingdao 266525, China; 3 School of National Safety and Emergency Management, Beijing Normal University, Beijing 100875, China

**Keywords:** real-time clearing price, interval prediction, feature selection, signal decomposition

## Abstract

With the rapid integration of renewable energy and the ongoing advancement of electricity market, accurate real-time prediction of electricity clearing prices has become increasingly critical. This paper introduces a deep learning-based framework for predicting real-time clearing price intervals, which employs time-shift feature construction and a greedy-based feature selection process to optimize high-dimensional feature sets. To address the nonlinear and non-stationary nature of price data, signal decomposition techniques are integrated to improve the extraction of underlying feature patterns. A point prediction model is first developed using a long short-term memory (LSTM) network, after which the upper and lower bounds of the prediction interval are derived via a similarity-based matching algorithm and statistical interval analysis, thereby explicitly quantifying market uncertainty. Numerical experiments based on the Shanxi Province (China) electricity market dataset show that the proposed model achieves higher interval coverage compared to baseline intervals, while maintaining practically useful interval widths, which offering more reliable decision support for electricity market participants.

## Highlights

• A novel electricity price interval prediction model is proposed
• Time-shift FC and greedy FS optimize electricity price feature dimensions
• VMD and CEEMDAN resolve extreme non-stationarity in clearing prices
• Non-parametric interval breaks coverage-sharpness trade-off in quantile regression

## Graphical abstract

**Figure:**
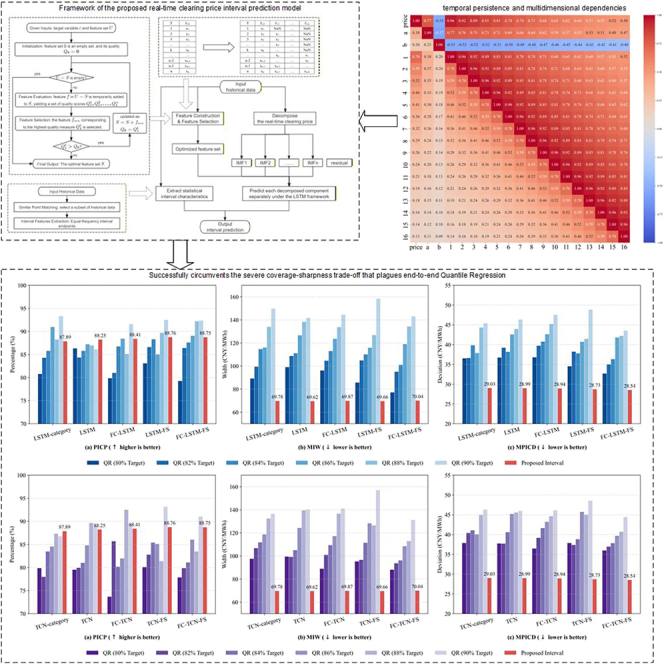

## Teaser

Energy policy; Economics

## Introduction

The real-time clearing price in electricity markets critically influences the economic interests of all market participants, rendering its accurate prediction vital for generation companies, consumers, and regulatory bodies. Existing research on electricity price forecasting is broadly divided into two methodological streams. The first involves simulating the power market to derive real-time clearing prices based on physical system configurations, including network topology, transmission line statuses, and unit dispatch conditions; however, such simulation-based methods require highly detailed and often inaccessible real-time system parameters, limiting their practicality for most market participants. In contrast, the second and more widely adopted approach leverages statistical and data-driven techniques to identify pricing patterns directly from historical market data, offering a feasible alternative that does not depend on full system observability.

Within the data-driven paradigm, methodologies can be categorized into three principal strands: (1) classical time series models, (2) machine learning and deep learning approaches, and (3) hybrid or ensemble techniques that integrate multiple models. Classical time series analysis, exemplified by autoregressive integrated moving average (ARIMA)^1^ and generalized autoregressive conditional heteroscedasticity (GARCH) models,^2^^,^^3^ seeks to capture the temporal dynamics of real-time clearing prices by exploiting their inherent dependencies. Nevertheless, these models are predominantly confined to a linear framework, which inherently restricts their capacity to represent the complex nonlinear interactions between price signals and their determinants.^4^ Consequently, they frequently exhibit limited predictive accuracy in the face of highly volatile price movements, with performance further hampered by difficulties in parameter selection and model specification.

Meanwhile, the advancement of machine learning and deep learning has propelled neural network-based methods to the forefront of real-time clearing price prediction. Techniques such as support vector machines (SVMs), random forests (RFs), recurrent neural networks (RNNs), long short-term memory (LSTM), and gated recurrent units (GRUs) have demonstrated significant potential in enhancing predictive performance. Real-time clearing prices are shaped by a complex interplay of factors, including power demand, meteorological conditions, as well as the positions of power units, which complicate accurate forecasting using any single modeling approach. This challenge has spurred the widespread adoption of hybrid and ensemble learning strategies.^5^^,^^6^ For instance, Shah et al.^7^ integrated neural networks with decision trees within an ensemble framework; Bibi et al.^8^ improved predictive accuracy by aggregating outputs from multiple heterogeneous models; and Kuo and Huang^9^ combined convolutional and LSTM networks to leverage both localized feature extraction and the capture of long-term temporal dependencies.

The variables mentioned above not only exist in large numbers but also interact through highly nonlinear and dynamically coupled relationships. To address this complexity, researchers have increasingly incorporated feature selection (FS) techniques into real-time price forecasting models to enhance predictive performance. For instance, Kapoor and Wichitaksorn^3^ integrated FS within a GARCH framework, demonstrating its effectiveness in improving accuracy in the New Zealand market. Similarly, Pourdaryaei et al.^10^ employed mutual information and neural network-based FS in a CNN-self-attention model to reduce input dimensionality and boost performance. Memarzadeh and Keynia^11^ introduced a hybrid model using entropy and mutual information to rank and eliminate redundant features, while Li and Becker^12^ emphasized the value of FS in LSTM models under market coupling conditions in Germany. Other approaches, such as the two-stage mutual information method combined with a wavelet-fuzzy system by Gao et al.,^13^ and the extra tree classifier with Pearson’s correlation used by Shafi et al.^14^ in a smart grid context, have also consistently shown that strategic FS contributes significantly to improving prediction accuracy.

Moreover, electricity market clearing prices exemplify non-stationary and nonlinear time series, which pose significant challenges to forecasting accuracy. To address these issues, signal decomposition techniques, such as empirical mode decomposition (EMD) and variational mode decomposition (VMD), have been widely adopted as essential preprocessing tools. For instance, Syah et al.^15^ applied fractional wavelet transform to decompose price data into high- and low-frequency components, optimizing predictions via a SVM, while Shafie-Khah et al.^16^ combined wavelet transform with ARIMA and radial basis function networks to capture both linear and nonlinear patterns. Gabrielli et al.^17^ introduced a data-driven method using Fourier analysis to separate price trends from volatile residuals, thereby improving long-term prediction across different markets. Similarly, Khan et al.^18^ proposed a three-stage framework integrating ensemble EMD with an extreme learning machine to enhance short-term forecasts, and Meng et al.^19^ employed empirical wavelet transform to preprocess inputs, reducing autocorrelation and boosting performance in renewables-rich markets. Further advancing the approach, Huang et al.^20^ and Yang et al.^21^ demonstrated that VMD significantly elevates prediction accuracy by adaptively extracting intrinsic mode functions (IMFs), with the former integrating VMD with LSTM and GRU networks, and the latter employing an adaptive parameter-based VMD to handle complex temporal structures effectively.

Despite the success of point prediction methods in electricity market forecasting, the offering of only a single best estimate makes them unable to quantify predictive uncertainty driven by the extreme volatility of electricity markets, which has driven growing interest in interval prediction. Interval prediction which provides upper and lower bounds at a specified confidence level to better inform risk assessment. Traditional interval forecasting approaches, such as bootstrap quantile regression (QR),^22^ kernel density estimation,^23^ and Gaussian processes,^24^ are theoretically sound but suffer from restrictive distributional assumptions and high computational costs for large-scale datasets. To overcome these limitations, non-parametric and deep learning-based alternatives have emerged, including error prediction methods,^25^ end-to-end QR,^26^^,^^27^ and conformal prediction (CP).^28^ However, constructing reliable prediction intervals for real-time electricity prices, characterized by extreme volatility, non-stationarity, and complex multidimensional dependencies, remains a formidable challenge.

Despite the success of point prediction methods in electricity market forecasting, the offering of only a single best estimate makes them unable to quantify predictive uncertainty driven by the extreme volatility of electricity markets, which has driven growing interest in interval prediction. Interval prediction provides upper and lower bounds at a specified confidence level to better inform risk assessment. Traditional interval forecasting approaches, such as bootstrap QR,^22^ kernel density estimation,^23^ and Gaussian processes,^24^ are theoretically sound but suffer from restrictive distributional assumptions and high computational costs for large-scale datasets. To overcome these limitations, non-parametric and deep learning-based alternatives have emerged, including error prediction methods,^25^ end-to-end QR,^26^^,^^27^ and CP.^28^ Furthermore, state-of-the-art (SOTA) temporal architectures, such as Temporal Convolutional Networks (TCNs),^29^ have been increasingly deployed in time series forecasting due to their causal convolutions and stable gradients, serving as powerful parametric baselines for uncertainty quantification when combined with QR. However, constructing reliable prediction intervals for real-time electricity prices, characterized by extreme volatility, non-stationarity, and complex multidimensional dependencies, remains a formidable challenge. Specifically, even for SOTA parametric models, using a global optimization function (e.g., pinball loss) invariably forces the network into a severe trade-off between coverage probability and interval sharpness when attempting to encapsulate extreme local price spikes.

**Figure 1:**
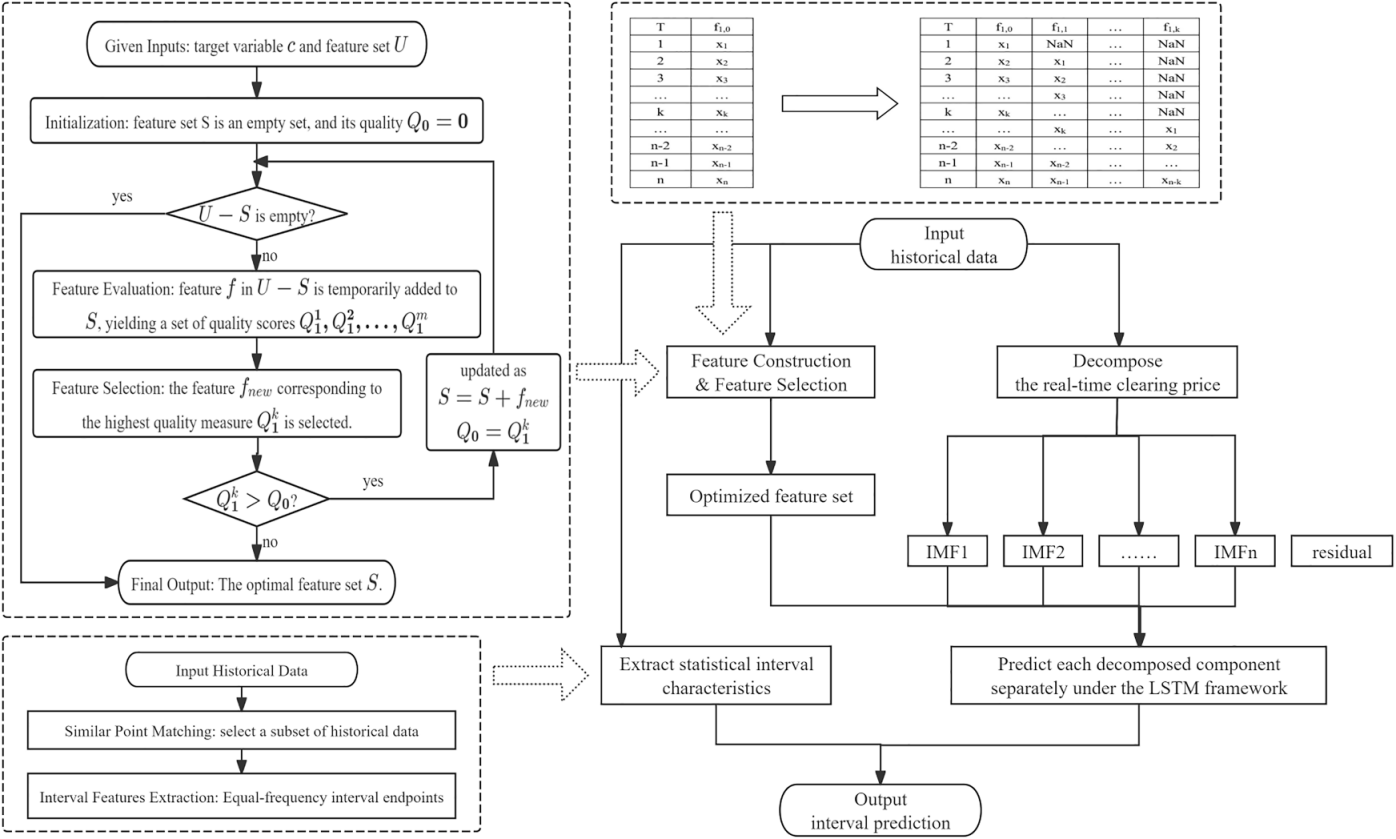
Framework of the proposed real-time clearing price interval prediction model Notes: The feature selection phase employs a greedy forward selection strategy, sequentially adding features to an initially empty set until an optimal combination is identified.

Motivated by these considerations, this paper proposes a novel data-driven framework for interval prediction of real-time electricity market clearing prices. The overall architecture is depicted in Figure 1. To establish a highly accurate baseline, the framework first constructs a robust point prediction model. This foundation employs a dedicated feature construction (FC) and selection pipeline to filter high-dimensional, noisy data, alongside signal processing techniques to transform non-stationary price series into stable IMFs for LSTM-based^30^^,^^31^ forecasting. Building upon this reliable point prediction, the framework focuses on a distribution-free interval construction module to quantify market uncertainty. Specifically, a cosine similarity-based matching algorithm is designed to identify a subset of historical periods that share key market and environmental characteristics with the target prediction point. By extracting empirical statistical variability (e.g., standard deviation, equal-frequency interval endpoints) from this matched historical subset and integrating it with the point forecast, the model generates calibrated upper and lower bounds.

By integrating these components, the proposed framework offers three key methodological advantages. First, market dimensionality and data redundancy are systematically addressed through a time-shift-based FC and greedy selection scheme, ensuring model parsimony and practical applicability without compromising predictive power. Second, the embedding of signal decomposition techniques, specifically, EMD, complete ensemble EMD with adaptive noise (CEEMDAN), or VMD, significantly mitigates the inherent non-stationarity of raw price sequences, effectively reducing the learning difficulty for subsequent neural networks. Finally, the novel nonparametric interval prediction architecture bridges accurate LSTM point forecasts with similarity-based empirical distributions. This synergy yields high-coverage, dynamically adaptive prediction intervals that provide actionable risk assessments for market participants under varying levels of uncertainty.

## Results

### Statistical and temporal characteristics of the data

We utilize real operational data from the Shanxi Province, China electricity market, covering the period from January 1, 2022, to July 1, 2024, with a 15-min temporal resolution (96 intervals per day). The dataset comprehensively characterizes the market through 16 initial features, including non-market unit generation, renewable energy output, and the target variable, the real-time clearing price. To enhance temporal representation, the timestamp was decomposed into three cyclical features: month, day, and intraday time index. Furthermore, recognizing the significant influence of weather on market dynamics, key environmental factors, i.e., wind speed, temperature, and precipitation, were incorporated as independent variables, sourced from the publicly available ERA5 dataset: https://cds.climate.copernicus.eu/datasets.

**Figure 2:**
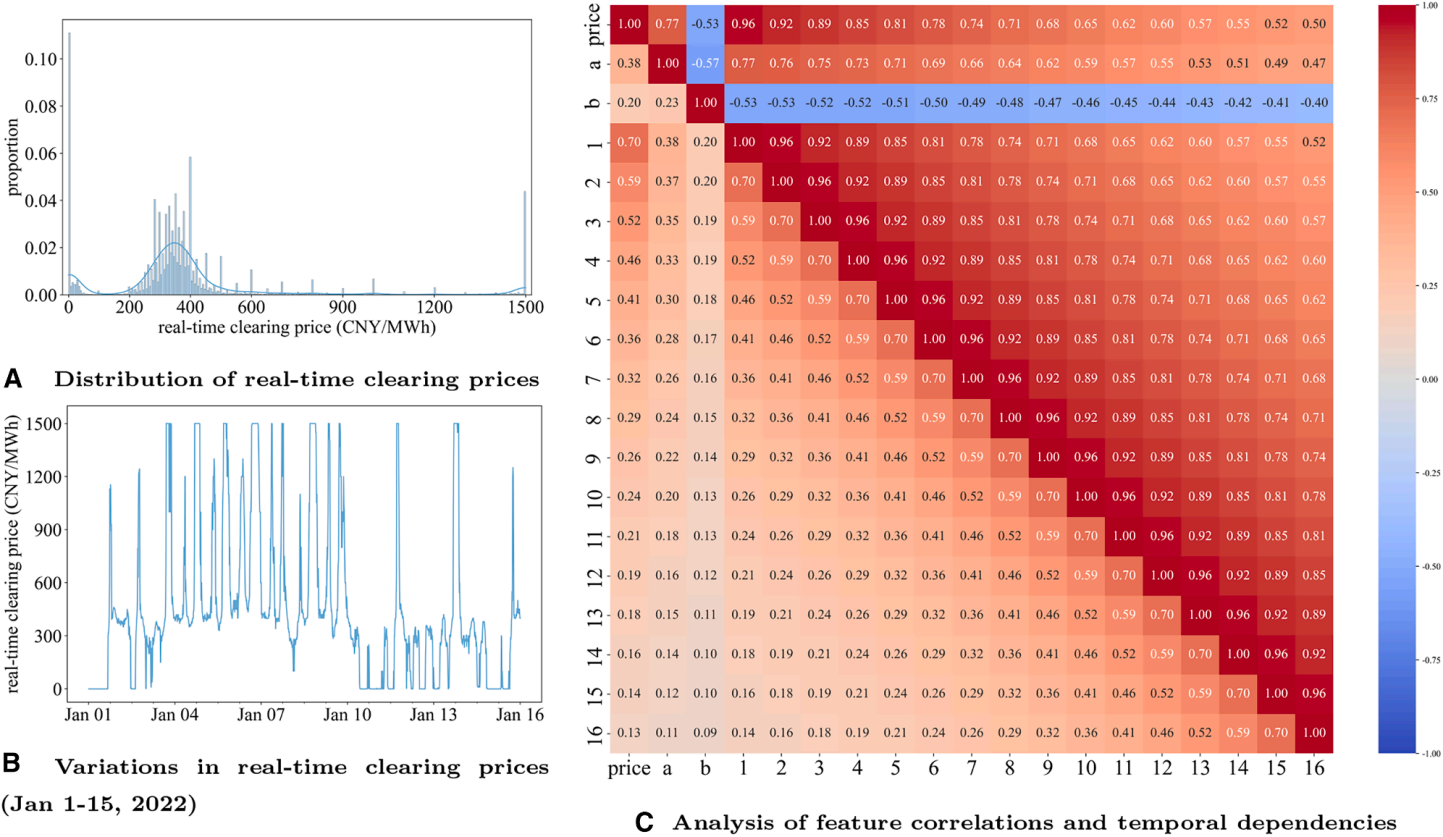
Characteristics of the real-time clearing price data (A) Price distribution histogram, showing concentration and extreme values. (B) Exemplary time series of price volatility over a two-week period. (C) Hybrid correlation matrix where the upper right triangle indicates Pearson’s correlation coefficients and the lower left triangle indicates symmetric uncertainty values. Here, *price* is the real-time clearing price, *a* is the day-ahead clearing price, *b* is renewable energy output, and *1 to 16* denotes the real-time price lagged by 1–16 periods respectively.

Figure 2 visualizes the statistical and temporal characteristics of the real-time clearing price. The price distribution is predominantly concentrated between 200 and 600 CNY/MWh, approximating a normal distribution within this interval (Figure 2A). However, notable frequencies of extreme values occur near the boundaries (around 0 and 1500 CNY/MWh), indicating occasional sharp price spikes and drops. This volatility is further illustrated in Figure 2B, which reveals pronounced fluctuations during the first half of January 2022. These patterns underscore the challenge of accurate price forecasting in a market that remains susceptible to significant outliers despite its general stability. To comprehensively evaluate the complex dependencies between features, Figure 2C presents a hybrid correlation matrix. The upper right triangle displays Pearson’s correlation coefficients to capture linear relationships, while the lower left triangle presents symmetric uncertainty (SU) values to quantify non-linear information dependencies. Both metrics consistently indicate that real-time clearing prices exhibit strong temporal persistence, particularly within the preceding 12 periods (3 h), highlighting the series’ short-term memory. The analysis further confirms the substantial impact of day-ahead prices and the significant negative correlation with renewable energy output. This affirms that recent historical data and these key market features provide considerable predictive power, justifying the use of sequence-based models such as LSTM to capture these dynamics.

To deeper investigate the structural market regimes driving these correlations, particularly the profound impact of renewable energy, we utilize the K-means algorithm^32^ to perform clustering analysis. The optimal number of clusters is determined by maximizing the silhouette coefficient.^33^ The analysis identifies two clusters as optimal, effectively segmenting the electricity market into two distinct operational regimes, with the distribution of key market characteristics for each cluster detailed in Table 1. The results reveal pronounced differences between the two regimes: Cluster 1 exhibits a significantly lower real-time clearing price (200 CNY/MWh) compared to Cluster 2 (501 CNY/MWh). While electricity load remains relatively consistent across both clusters, their supply-side structures differ markedly. Cluster 1 is characterized by substantially higher renewable energy output and slightly lower non-market unit generation, whereas Cluster 2 shows heavy reliance on non-market units, coinciding with its higher market price. These findings suggest that renewable energy penetration is a primary driver of real-time price formation, as the abundance of renewable generation in Cluster 1 appears to displace more expensive, non-market unit generation, thereby reducing clearing prices. However, the inherent variability of wind and photovoltaic power introduces significant volatility. This non-dispatchable generation profile complicates real-time price fluctuations, posing challenges for both stable market operations and accurate price forecasting.

**Table 1:** Distribution of electricity market characteristics across clusters

Characteristic	Overall dataset	Cluster 1	Cluster 2
Number of samples	87996 (100.00%)	30819 (35.02%)	57177 (64.98%)
Real-time clearing price (CNY/MWh)	396 (327)	200 (178)	501 (341)
Non-market unit generation (MW)	3616 (3098)	2703 (2552)	4108 (3250)
Renewable energy output (MW)	7643 (4545)	12592 (3150)	4975 (2640)
Electricity load (MW)	27424 (3153)	27019 (3324)	27643 (3036)

Values represent mean (standard deviation).

### Point prediction performances

To evaluate the generality and effectiveness of the proposed FC and selection pipeline, we initially establish four classical regression models (Linear Regression,^34^ Decision Tree,^35^ Bagging Tree,^36^ and Random Forest^37^; detailed results are provided in Supplemental Information S1). The analysis yielded two key insights: first, FC significantly enhances the ability of non-sequential models to capture temporal dynamics; second, FS consistently reduces model dimensionality without compromising predictive accuracy. Building upon these findings, we deployed the LSTM architecture, uniquely suited for sequential data, configured with an input sequence length of one day (96 historical intervals) to capture relevant temporal dependencies. As summarized in Table 2, integrating clustering labels into the LSTM yielded no significant improvements, indicating that the network inherently captures market regime heterogeneities. Furthermore, because LSTM’s recurrent gates naturally model temporal dependencies, the time-shift-based FC offered only marginal gains. In contrast, the FS approach retained its high utility, successfully streamlining the input dimensions while maintaining predictive accuracy, thereby enhancing computational parsimony for real-time applications.

**Table 2:** LSTM model performance under different feature preprocessing methods

Model	R-squared	RMSE	MAE	MAPE	SMAPE
LSTM-category	0.8696	88.36	27.2202	10.36%	8.29%
LSTM	0.8696	88.357	27.3845	10.38%	8.25%
FC-LSTM	0.8683	88.7905	27.2048	10.52%	8.33%
LSTM-FS	0.8697	88.3377	26.9728	10.63%	8.37%
FC-LSTM-FS	0.8695	88.4073	26.6371	10.17%	8.09%
LSTM-FS-EMD	0.9389	60.4851	27.6631	12.05%	12.05%
FC-LSTM-FS-EMD	0.936	61.9284	32.1745	13.97%	12.07%
LSTM-FS-CEEMDAN	0.9553	51.7176	23.5386	10.31%	10.27%
FC-LSTM-FS-CEEMDAN	0.9556	51.5585	22.5425	9.86%	9.18%
LSTM-FS-VMD	0.9446	57.6017	26.1388	10.94%	9.83%
FC-LSTM-FS-VMD	0.947	56.3282	24.2311	10.34%	8.76%

(a) The base LSTM model uses 22 input features. This is expanded to 50 with FC, reduced to 17 with FS, and optimized to 16 with the combined FC-FS method. (b) For signal decomposition, EMD and CEEMDAN were implemented using the default parameters of the standard Python libraries. For VMD, the empirical parameters were configured as follows to ensure optimal decomposition and reproducibility: number of modes *K* = 5, bandwidth constraint *α* = 2000, and noise tolerance *τ* = 0. (c) To strictly prevent information leakage, datasets were partitioned chronologically. The network comprises two stacked LSTM layers (64 and 16 hidden units) with a 0.05 dropout rate, culminating in a fully connected layer. Models were trained via the Adam optimizer (learning rate 10^−3^), utilizing dynamic shuffling for training batches and an Early Stopping mechanism (patience = 10) to restore optimal validation weights. Standard Min-Max normalization was applied to stabilize network convergence, while the evaluation metrics were computed after reconstructing back to their original scale.

To further address the severe non-stationarity of the price series, EMD and its advanced variants, CEEMDAN and VMD, were integrated into the LSTM-FS framework. As evident in Table 2, there is a clear performance divergence. While standard EMD improves global accuracy, its inherent mode mixing deteriorates certain metrics. Conversely, CEEMDAN (via adaptive noise addition) and VMD (via variational band-pass filtering) effectively isolate distinct frequency components without recursive sifting. Consequently, both CEEMDAN and VMD significantly outperform the undecomposed baseline, yielding highly competitive global enhancements, particularly driving R-squared above 0.94 and drastically reducing RMSE.

However, a critical trade-off emerges when examining relative error metrics (MAPE and SMAPE). Advanced decomposition methods act as sophisticated smoothing filters, stripping away extreme, high-frequency market noise to capture continuous primary trends. While this effectively minimizes large absolute deviations, it inevitably alters the original joint probability distribution and suppresses the jagged variance of the raw price series. Because relative errors are hypersensitive to minor absolute deviations in low-price regimes, the reconstructed, smoothed signals can occasionally inflate percentage-based errors. Despite this localized inflation, the overwhelming gains in global stability confirm that the synergistic integration of sequence modeling (LSTM), streamlined inputs (FS), and advanced signal decomposition (CEEMDAN/VMD) constitutes a highly accurate and robust point prediction core.

### Uncertainty quantification via interval prediction

To operationalize the proposed interval construction framework and rigorously benchmark its performance, specific piecewise algorithmic rules and comparative baselines were established. Briefly, the proposed nonparametric generator takes the LSTM point prediction as input and maps it to an interval, meaning the quality of the interval fundamentally depends on the accuracy of the point prediction. The interval bounds are adaptively scaled according to empirical quantile bins derived from a pre-constructed reference set of historically similar patterns (identified via cosine similarity matching), strictly adhering to explicit market regulatory constraints (e.g., maximum price ceilings). Detailed algorithmic parameters and the piecewise interval integration logic are comprehensively documented in the Supplemental Information S2. To rigorously assess its efficacy, the model is evaluated against four probabilistic paradigms: (1) a naive error-based reference interval; (2) an end-to-end LSTM-based QR (LSTM-QR) model systematically swept across 80%–90% nominal coverages to assess the coverage-sharpness trade-off; (3) a TCN combined with QR (TCN-QR), representing the current SOTA parametric probabilistic approach; and (4) CP, whose significance level is dynamically calibrated to match the empirical coverage of the proposed model, ensuring a strictly unbiased comparison of interval sharpness. Specific configuration details for these baselines are provided in the Supplemental Information S3.

**Figure 3:**
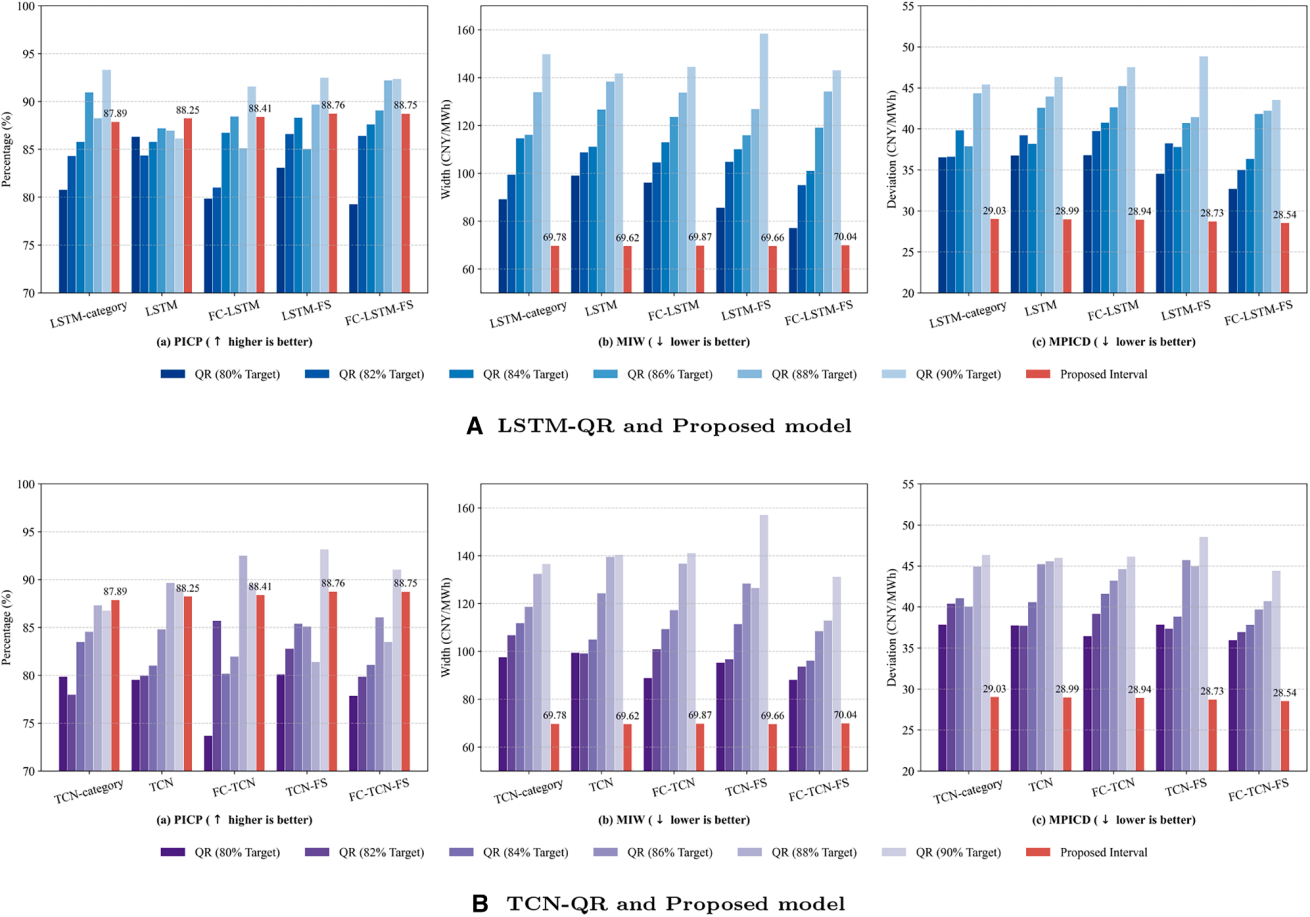
Interval prediction metrics across 80%–90% targets compared with the proposed model Notes: The proposed interval metrics are labeled in each subplot to demonstrate their structural advantage. To explicitly investigate the parametric trade-off, the TCN-QR architecture in B was configured with multi-level causal temporal blocks (32, 64, and 128 hidden channels) and a kernel size of 3, featuring a 0.05 dropout rate. Both QR baselines optimized multi-stream quantile outputs directly via the pinball loss function. Models were trained using the Adam optimizer (learning rate 10^−3^) with dynamic batch shuffling. To strictly prevent information leakage, the early stopping mechanism (patience = 10) was applied on a chronologically partitioned validation set to restore optimal weights, and standard Min-Max normalization was executed with evaluations computed after inverse transformation.

The experimental results, presented in Table 3 and Figure 3, yield several crucial findings that robustly substantiate the efficacy and superiority of the proposed nonparametric empirical interval model. Initially examining the undecomposed model configurations, the proposed interval generation framework demonstrates a distinct structural advantage. Compared to the naive error-based baseline, the proposed model significantly improves the prediction interval coverage probability (PICP), elevating it from approximately 82%–83% to nearly 88%–89%. This indicates a markedly superior capability to encapsulate the true underlying distribution of price uncertainty. Crucially, this substantial gain in reliability is achieved while rigorously maintaining interval sharpness; the mean interval width (MIW) remains highly stable at approximately 69 CNY/MWh, and the mean prediction interval center deviation (MPICD) experiences only a negligible shift.

**Table 3:** Interval prediction model results under the LSTM framework

Model	Error-based (±10%)			Conformal prediction (CP)			Proposed interval		
	PICP	MIW	MPICD	PICP	MIW	MPICD	PICP	MIW	MPICD
LSTM-category	82.53%	69.23	27.22	88.05%	85.78	31.13	87.89%	69.78	29.03
LSTM	82.72%	68.99	27.38	87.51%	89.90	31.49	88.25%	69.62	28.99
FC-LSTM	83.45%	69.35	27.20	88.28%	86.81	31.07	88.41%	69.87	28.94
LSTM-FS	82.04%	69.22	27.05	88.19%	85.83	30.92	88.76%	69.66	28.73
FC-LSTM-FS	82.97%	69.51	26.64	88.13%	82.38	30.40	88.75%	70.04	28.54
LSTM-FS-EMD	75.92%	69.45	27.66	79.97%	76.39	31.25	78.12%	73.31	29.89
FC-LSTM-FS-EMD	73.34%	71.92	32.17	77.77%	81.80	35.30	76.62%	76.32	33.83
LSTM-FS-CEEMDAN	77.78%	69.84	23.54	79.57%	71.31	26.85	80.60%	73.60	26.22
FC-LSTM-FS-CEEMDAN	78.91%	69.87	22.54	81.10%	72.45	25.51	81.61%	73.79	25.17
LSTM-FS-VMD	78.19%	67.94	26.14	79.91%	72.48	29.92	80.74%	73.71	29.03
FC-LSTM-FS-VMD	79.59%	69.24	24.23	82.67%	77.58	27.92	82.87%	75.17	27.00

Optimal prediction intervals should achieve high coverage (PICP) while maintaining narrow width (MIW) and small deviation (MPICD).

When contrasted with both parametric QR paradigms (LSTM-QR and TCN-QR), the proposed model successfully breaks the severe trade-off between coverage probability and interval sharpness. As illustrated in Figure 3, systematically pushing the pinball loss function to achieve higher theoretical coverages exposes a fundamental limitation of parametric models: to capture sudden extreme price spikes within a global optimization function, QR invariably stretches its intervals across all data points. Consequently, as the target coverage increases from 80% to 90%, the MIW for LSTM-QR drastically inflates from approximately 77-99 CNY/MWh to a staggering 141–158 CNY/MWh. Strikingly, even with the advanced causal feature extraction capabilities of TCN, the model cannot escape this parametric bottleneck; forcing TCN-QR to achieve a ∼90% coverage (e.g., PICP = 91.07% under the optimal FC-FS configuration) results in a heavily penalized MIW of 131.24 CNY/MWh and an MPICD of 44.46. Even at their most conservative settings (80% target), the MIWs of both QR models remain inferior to the proposed model. In stark contrast, by anchoring localized, historical bin-matching with regulatory-informed truncation rules, the proposed non-parametric model inherently bypasses this limitation. It achieves a highly competitive empirical coverage (∼88%) while solidly constraining the MIW to approximately 69 CNY/MWh, providing much sharper and actionable conditional coverage that end-to-end SOTA models struggle to replicate.

Furthermore, when compared against the dynamically calibrated CP, the proposed method exhibits superior adaptability to market heteroscedasticity. Standard CP constructs intervals by applying a global non-conformity threshold, which fundamentally forces a rigid, constant interval width across all forecasting points regardless of the immediate market state. This inevitably overestimates uncertainty during calm regimes and underestimates risk during price spikes. The proposed method resolves this by dynamically tailoring the interval width for each specific time step based on the matched historical bins and regulatory rules. As a result, even when aligned to the exact same PICP level, the proposed method consistently exhibits narrower MIW and superior center calibration (lower MPICD) than the CP baseline, generating highly adaptive bounds that accurately reflect the true dynamic risk profile of the electricity market.

Finally, when signal decomposition techniques, i.e., EMD, CEEMDAN, and VMD, are integrated, the proposed method proves highly resilient in mitigating the inherent statistical distortions caused by signal smoothing. As previously established, decomposition acts as a low-pass filter; while it vastly improves global point accuracy, it alters the original joint probability distribution, and the reconstructed smoothed signals occasionally struggle to align perfectly with raw low-price regimes, causing the coverage of naive error-based intervals to collapse precipitously to the 73%–79% range. Despite this altered probability distribution, the proposed nonparametric method actively recovers approximately 3 percentage points in PICP across various configurations. While this recovery naturally requires a modest widening of the MIW (by roughly 4–6 CNY/MWh), this is an entirely acceptable and necessary trade-off to ensure robust uncertainty bounds in a highly volatile market. Furthermore, its overall interval sharpness and calibration remain structurally competitive or superior to CP even under these complex decomposed conditions.

## Discussion

This study proposes a comprehensive, data-driven interval prediction framework for real-time electricity market clearing prices, meticulously designed to tackle the formidable challenges of extreme volatility, non-stationarity, and multidimensional market dependencies. Evaluated on an extensive real-world dataset from the Shanxi Province electricity market, the proposed architecture seamlessly integrates feature set optimization, advanced signal decomposition, and a novel nonparametric interval construction technique to deliver actionable uncertainty quantification.

The results indicate that feature set optimization, strategic combination of time-shift-based FC and greedy algorithm-based FS successfully balanced the extraction of complex temporal dependencies with vital model parsimony. Moreover, the integration of advanced signal decomposition techniques, particularly CEEMDAN and VMD, proved highly effective in mitigating price non-stationarity. These methodological contributions lead to superior predictive performance while maintaining model practical applicability.

Furthermore, the proposed nonparametric interval prediction methodology effectively rectifies the inadequacies of conventional probabilistic paradigms. By synergizing similarity-based historical distribution matching with explicit regulatory constraints, the model dynamically adapts to market heteroscedasticity. It successfully circumvents the severe coverage-sharpness trade-off that plagues end-to-end QR (which globally stretches intervals to capture local extremes) and overcomes the rigid, constant-width limitations of standard CP. Whether applied to raw, highly volatile price signals or navigating the suppressed variance inherent in decomposed sub-series, the proposed model consistently delivers sharp, highly calibrated prediction intervals that dynamically reflect the true risk profile of the market.

Overall, this study estab lishes a robust and highly adaptable framework for real-time clearing price prediction, offering substantial practical value for electricity market participants in risk assessment and decision-making. The findings underscore the potential of deep learning methods in capturing the complex dynamics of electricity prices and lay a foundation for future research. Subsequent work may explore the application of this framework in other electricity markets, incorporate external economic and policy factors, and investigate more advanced neural network architectures to further enhance predictive accuracy and reliability.

### Limitations of the study

The current feature set is restricted to internal market generation variables, historical prices, and basic meteorological data. It does not account for broader macroeconomic indicators, fuel price volatility (e.g., coal or natural gas prices), real-time physical transmission constraints (network congestion), or unexpected generator outages. The absence of these underlying physical and economic drivers limits the model’s ability to anticipate price variations caused by structural grid issues.

## Resource availability

### Lead contact

Further information and requests for resources should be directed to and will be fulfilled by the lead contact, Lei Zhu (leizhu@bnu.edu.cn).

### Materials availability

As this study did not generate new unique material, the material availability is not applicable.

### Data and code availability

The raw data are not publicly available due to confidentiality and licensing agreements regarding real-world electricity market operations, but can be obtained from the lead contact upon reasonable request. The code is openly accessible at https://github.com/xinyi-yj/Non-parametricIntervalPrediction. Any additional information required to reanalyze the data is also available from the lead contact upon request.

## Acknowledgments

We would like to thank the editor and the anonymous reviewers for their constructive comments to improve our article. This work was supported by the 10.13039/501100001809National Natural Science Foundation of China (grant nos. 72342002, 72122002, and 72394374).

## Author contributions

Supervision: L.Z.; conceptualization: J.Y. and L.Z.; methodology: J.Z. and J.Y.; data collection: L.Z.; writing – original draft: L.Z. and J.Y.; writing – review and editing: J.Y. and L.Z.; visualization: J.Y. J.Y. and J.Z. contributed equally to the paper.

## Declaration of interests

The authors declare no competing interests.

## STAR★Methods

### Key resources table

**Table:** 

REAGENT or RESOURCE	SOURCE	IDENTIFIER
**Deposited data**		

Shanxi Province electricity market dataset (Jan 1, 2022 to Jul 1, 2024)	This paper	Available upon reasonable request from the lead contact
ERA5 data	Copernicus Climate Change Service (C3S) Climate Data Store	https://cds.climate.copernicus.eu/datasets

**Software and algorithms**		

Custom code for Non-parametric Interval Prediction model	This paper	GitHub: https://github.com/xinyi-yj/Non-parametricIntervalPrediction
Python (Version 3.12)	Python Software Foundation	https://www.python.org/
PyTorch (Version 2.11.0+cu126)	PyTorch Foundation	https://pytorch.org/
Scikit-learn (Version 1.9.0)	Scikit-learn developers	https://scikit-learn.org/
EMD-signal (Version 1.9.0)	PyPI	https://pypi.org/project/EMD-signal/
vmdpy (Version 0.2)	PyPI	https://pypi.org/project/vmdpy/
NumPy (Version 2.4.6)	NumPy developers	https://numpy.org/
Pandas (Version 3.0.3)	Pandas development team	https://pandas.pydata.org/

### Method details

#### Feature optimization and data preprocessing

To capture the complex nonlinear dynamics driven by diverse market and environmental factors, this study initially constructs a high-dimensional feature space comprising 15 key internal market variables (including thermal, hydro, wind, and photovoltaic power generation outputs, interconnection power flows, provincial and regulated system loads, and day-ahead clearing prices) alongside three external environmental factors (temperature, wind speed, and precipitation). To mitigate the risks of overfitting and high computational cost associated with this initial high dimensionality, a two-stage preprocessing methodology comprising feature construction and feature selection is employed to derive an optimal, informative, and non-redundant feature subset.

The feature construction phase enhances the temporal representational capacity of the initial data. As illustrated in Figure 1, this is achieved through time-shift labeling, where an original feature *f*_*i*,0_ is expanded into a series of lagged variables *f*_*i*,1_, *f*_*i*,2_, *…*, *f*_*i*,*k*_. Here, *f*_*i*,*k*_ at time *T* corresponds to the value of *f*_*i*,0_ at time *T* − *k*. This method explicitly captures auto-regressive patterns and temporal dependencies, thereby providing the predictive model with a richer historical context.

Subsequently, feature selection is applied to this expanded candidate set to reduce dimensionality while preserving predictive power. An optimal feature set should maximize collective relevance to the target variable *c* (the real-time clearing price) while minimizing inter-feature redundancy.^38^ The quality *J*(*S*) of a candidate feature subset *S* is quantified by the objective function:

(Equation 1) $$J\left(S\right)=\bar{SU}\left(S,c\right)-\beta \bar{SU}\left(S,S\right)$$

(Equation 2) $$\bar{SU}\left(S,c\right)=\frac{\sum_{f_{i}\in S}SU\left(f_{i},c\right)}{m_{S}}$$

(Equation 3) $$\bar{SU}\left(S,S\right)=\frac{\sum_{f_{i}\in S}\sum_{f_{j}\in S}SU\left(f_{i},f_{j}\right)}{m_{S}^{2}}$$

where $\bar{SU}\left(S,c\right)$ represents the average symmetric uncertainty (SU) between features in *S* (which contains *m*_*S*_ features) and the target *c* (Equation 2), serving as a measure of relevance. Conversely, $\bar{SU}\left(S,S\right)$ denotes the average SU among features within *S* (Equation 3), quantifying redundancy. The trade-off parameter *β* balances the importance of these two components. The SU between any two variables *a* and *b* is defined as:

(Equation 4) $$SU\left(a,b\right)=2\;\cdot \;\frac{H\left(a\right)-H\left(a|b\right)}{H\left(a\right)+H\left(b\right)}$$

where *H*(⋅) denotes entropy and *H*(⋅|⋅) denotes conditional entropy.

To efficiently optimize *S*, a greedy forward selection algorithm is employed, as depicted in Figure 1. The algorithm initializes with an empty set and iteratively selects the feature from the candidate pool *U* that yields the maximum increase in *J*(*S*). This stepwise evaluation prioritizes features that are highly informative about the target yet exhibit low correlation with those already selected. The process continues until a predefined stopping criterion is met, resulting in a compact, high-quality feature set that effectively mitigates overfitting and enhances model generalizability and performance.

#### Signal decomposition of clearing price time series

The real-time clearing price series exhibits pronounced nonlinearity and non-stationarity, presenting a significant challenge for direct forecasting. To mitigate this, the original price series is decomposed into more stable and tractable sub-components using EMD, CEEMDAN, and VMD. These methods disentangle the complex price signal into IMFs with distinct frequency characteristics, thereby reducing data complexity and enhancing the predictive model’s ability to capture underlying patterns.

The EMD method adaptively decomposes a signal into oscillatory components based on its local characteristics.^39^ For a given real-time clearing price series *P*(*t*), the iterative sifting process is as follows:

*Step 1*, Identify Envelopes and Mean: Identify all local extrema of *P*(*t*). Construct the upper envelope *P*_*up*_(*t*) and lower envelope *P*_*low*_(*t*) by connecting the maxima and minima, respectively, using cubic spline interpolation. The mean envelope is then calculated as:

(Equation 5) $$m_{1}\left(t\right)=\frac{P_{up}\left(t\right)+P_{\mathrm{low}}\left(t\right)}{2}$$

*Step 2*, Extract the Detail Component: Subtract the mean envelope from the signal to obtain a candidate component:

(Equation 6) $$h_{1}\left(t\right)=P\left(t\right)-m_{1}\left(t\right)$$

Check if *h*_1_(*t*) satisfies the criteria of an IMF (number of extrema and zero-crossings differs at most by one; mean of its envelope is zero). If not, treat *h*_1_(*t*) as the new signal and repeat Steps 1–2 iteratively until the criteria are met. The resulting component is designated as the first IMF, *C*_1_(*t*).

*Step 3*, Calculate the Residual: Separate the first IMF from the original signal to obtain the residual:

(Equation 7) $$r_{1}\left(t\right)=P\left(t\right)-C_{1}\left(t\right)$$

*Step 4*, Iterate the Process: Treat the residual *r*_1_(*t*) as the new data series and repeat the entire sifting process (steps 1–3) to subsequent IMFs *C*_2_(*t*), *C*_3_(*t*), *…*, *C*_*n*_(*t*). The process concludes when the residual *r*_*n*_(*t*) becomes a monotonic function from which no more IMFs can be extracted. The original real-time clearing price series is thus reconstructed as:

(Equation 8) $$P\left(t\right)=\overset{n}{\underset{i=1}{\sum }}C_{i}+r_{n}\left(t\right)$$

Although EMD is highly adaptive, it is inherently susceptible to the mode mixing problem, where signals of disparate scales manifest within a single IMF, or a single scale is distributed across multiple IMFs due to intermittency in the real-time clearing price series. To address this limitation, CEEMDAN is incorporated into our methodology as an advanced variant.^40^ CEEMDAN improves upon EMD and traditional ensemble EMD (EEMD) by adding a finite amount of adaptive white noise at each stage of the decomposition rather than just to the original signal. By extracting the unique residue and calculating the ensemble mean of the local means at each stage, CEEMDAN effectively isolates the true intrinsic modes. This mechanism not only resolves the mode mixing issue but also ensures a complete and exact reconstruction of the original signal with near-zero reconstruction error.

Meanwhile, VMD is a non-recursive, variational framework that decomposes a signal by solving a constrained optimization problem.^41^ It is particularly effective at mitigating mode mixing and offers greater robustness than EMD. The VMD algorithm seeks to decompose the real-time clearing price series *P*(*t*) into *K* predefined modes, *u*_*k*_(*t*), each with a limited bandwidth around a center frequency *ω*_*k*_. The process is as follows:paragraph*Step 1*, Initialization: Initialize the mode signals ${\left\{u_{k}\left(t\right)\right\}}_{k=1}^{K}$ and their center frequencies ${\left\{\omega_{k}\right\}}_{k=1}^{K}$. Define the parameters: number of modes *K*, bandwidth constraint *α*, penalty factor *λ* and tolerance *ϵ*.

*Step 2*, Mode and Frequency Update: For all *k* = 1, *…*, *K*, update the mode signal *u*_*k*_(*t*) and its center frequency *ω*_*k*_ for the current iteration while keeping other modes fixed. The mode signal update is performed by minimizing the following objective function:

(Equation 9) $$u_{k}\left(t\right)=\arg\;\underset{u_{k}}{\min}\left[\alpha {\left\|\partial_{t}\left(\left[X\left(t\right)-\underset{i\neq k}{\sum }u_{i}\left(t\right)\right]e^{-j\omega_{k}t}\right)\right\|}^{2}+\lambda {\left\|\nabla u_{k}\right\|}^{2}\right]$$

where *j* is the imaginary unit. After updating the model signal, updates its center frequency as:

(Equation 10) $$\omega_{k}=\frac{\int t|u_{k}\left(t\right){|}^{2}dt}{\int |u_{k}\left(t\right){|}^{2}dt}$$

*Step 3*, Convergence Check: Compute the variation in mode signals and center frequencies. If the variation is smaller than the tolerance *ϵ*, the algorithm terminates. Otherwise, repeat step 2 for further refinement.

*Step 4*, Output of IMFs: Once convergence conditions are met, each mode *u*_*k*_(*t*) is regarded as a finite-bandwidth IMF. The original real-time clearing price series *P*(*t*) is reconstructed as the sum of all mode signals and a residual component:

(Equation 11) $$P\left(t\right)=\overset{K}{\underset{i=1}{\sum }}u_{k}\left(t\right)+r_{n}\left(t\right)$$

where *r*_*n*_(*t*) is the final residual component.

#### Nonparametric interval construction

To quantify the inherent uncertainty in real-time clearing prices, a nonparametric interval prediction module is constructed to estimate upper and lower bounds without restrictive distributional assumptions, offering a probabilistic view of potential fluctuations. This provides market participants with a more robust foundation for risk assessment and strategic decision-making. As illustrated in Figure 1, this process comprises three sequential steps.

First, Similar Point Matching is performed to identify a relevant historical context for the target forecast point. A cosine similarity-based algorithm is employed to compute the similarity between the current market state (defined by a vector of key market and environmental features) and all historical instances. A subset of the most similar historical points is then selected, forming a reference group that shares analogous market and environmental conditions.

Second, Statistical Characterization is conducted on the selected similar-point subset. The empirical distribution of the actual real-time clearing prices within this subset is analyzed to extract key statistical metrics. These metrics, which include measures of central tendency (e.g., mean), dispersion (e.g., standard deviation), and equal-frequency interval endpoints, are used to define the shape and variability of the expected price distribution.

Finally, Interval Integration combines the statistical characteristics from the similar-point subset with the point forecast. The point prediction, generated by the core LSTM model, provides the a single best estimate. This estimate is then integrated with the dispersion and equal-frequency interval information from the similar points to construct the final prediction intervals. For instance, the point forecast within an interval whose bounds are determined by the historical volatility observed under similar conditions.

By leveraging this three-step framework, the constructed prediction intervals effectively encapsulate both the expected price level and its associated uncertainty. This offers a more comprehensive representation of potential market outcomes, ultimately enhancing risk management and supporting more informed trading decisions.

### Quantification and statistical analysis

All statistical analyses, data preprocessing, and deep learning model training were performed using Python (version 3.12) with relevant libraries including NumPy, Pandas, Scikit-learn for basic data processing, and PyTorch for LSTM network implementation. To quantitatively assess the model’s performance, a comprehensive evaluation framework incorporating metrics for both point and interval prediction was employed. The full suite of metrics and their corresponding statistical formulas applied in this study is detailed in Table 4

**Table 4:** Evaluation metrics for point and interval prediction

Metric	Formula
**Point Prediction Evaluation**	

Coefficient of Determination (*R*^2^)	$R^{2}=1-\frac{\sum_{i=1}^{n}{\left(y_{i}-\hat{y_{i}}\right)}^{2}}{\sum_{i=1}^{n}{\left(y_{i}-\bar{y}\right)}^{2}}$
Root Mean Squared Error (RMSE)	$RMSE=\sqrt{\frac{1}{n}\sum_{i=1}^{n}{\left(y_{i}-\hat{y_{i}}\right)}^{2}}$
Mean Absolute Error (MAE)	$MAE=\frac{1}{n}\sum_{i=1}^{n}|y_{i}-\hat{y_{i}}|$
Mean Absolute Percentage Error (MAPE)	$MAPE=\frac{100}{n}\sum_{i=1}^{n}\left|\frac{y_{i}-\hat{y_{i}}}{y_{i}}\right|$
Symmetric Mean Absolute Percentage Error (SMAPE)	$SMAPE=\frac{100}{n}\sum_{i=1}^{n}\frac{2\times |y_{i}-\hat{y_{i}}|}{\left|y_{i}|+|\hat{y_{i}}\right|}$

**Interval Prediction Evaluation**	

Prediction Interval Coverage Probability (PICP)	$PICP=\frac{1}{n}\sum_{i=1}^{n}I\left(y_{i}\in \left[l_{i},u_{i}\right]\right)$
Mean Interval Width (MIW)	$MIW=\frac{1}{n}\sum_{i=1}^{n}\left(u_{i}-l_{i}\right)$
Mean Prediction Interval Center Deviation (MPICD)	$MPICD=\frac{1}{n}\sum_{i=1}^{n}\left(y_{i}-\frac{l_{i}+u_{i}}{2}\right)$

*y*_*i*_ and $\hat{y_{i}}$ are the actual and predicted values; $\bar{y}$ is the mean of actual values; *l*_*i*_ and *u*_*i*_ denote the lower and upper bounds of the prediction interval; *I*(⋅) is an indicator function that returns 1 if *y*_*i*_ ∈ [*l*_*i*_, *u*_*i*_] and 0 otherwise.

.

For point prediction, a suite of metrics is used to evaluate different aspects of performance. The coefficient of determination (*R*^2^) quantifies the proportion of variance in the actual data explained by the model, with values closer to 1 indicating a superior fit. The root mean squared error (RMSE) measures the standard deviation of prediction errors, heavily penalizing larger deviations due to its quadratic nature, which is critical in precision-sensitive applications like price forecasting. In contrast, the mean absolute error (MAE) provides a more interpretable and robust measure of the average error magnitude. To evaluate relative accuracy, the mean absolute percentage error (MAPE) is considered, though it can be unstable for values near zero. The symmetric mean absolute percentage error (SMAPE) mitigates this issue by normalizing the error against the average of the actual and predicted values, providing a more balanced percentage error.^42^

For interval prediction, the evaluation focuses on reliability and informativeness. The prediction interval coverage probability (PICP) measures the empirical coverage rate, i.e., the proportion of actual observations that fall within the predicted intervals. A higher PICP indicates better reliability, but it can be trivially achieved by generating excessively wide intervals. Therefore, the mean interval width (MIW) is reported alongside to quantify the average sharpness of the intervals. The ideal model achieves a high PICP with a narrow MIW. Finally, the mean prediction interval center deviation (MPICD) assesses the calibration of the interval’s center by measuring its average deviation from the actual value, ensuring the intervals are correctly positioned.^43^

By integrating these complementary metrics (summarized in Table 4), the model’s predictive accuracy and its capacity to represent uncertainty can be systematically validated, ensuring its practical utility for decision-making in real-time electricity markets.

## References

[1] Popovska E., Georgieva-Tsaneva G. (2022). Arima model for day-ahead electricity market price forecasting. Science Series-Innovative STEM Education.

[2] Godekere P.G. (2016). Spot electricity price forecasting in indian electricity market using autoregressive-garch models. Energy Strategy Rev..

[3] Kapoor G., Wichitaksorn N. (2023). Electricity price forecasting in new zealand: A comparative analysis of statistical and machine learning models with feature selection. Appl. Energy.

[4] Lago J., De Ridder F., De Schutter B. (2018). Forecasting spot electricity prices: Deep learning approaches and empirical comparison of traditional algorithms. Appl. Energy.

[5] Chang Z., Zhang Y., Chen W. (2019). Electricity price prediction based on hybrid model of adam optimized lstm neural network and wavelet transform. Energy.

[6] Zhang J., Tan Z., Wei Y. (2020). An adaptive hybrid model for short term electricity price forecasting. Appl. Energy.

[7] Shah R., Shah H., Bhim S., Heistrene L., Pandya V. (2021). 2021 1st International Conference in Information and Computing Research (iCORE).

[8] Bibi N., Shah I., Alsubie A., Ali S., Lone S.A. (2021). Electricity spot prices forecasting based on ensemble learning. IEEE Access.

[9] Kuo P.-H., Huang C.-J. (2018). An electricity price forecasting model by hybrid structured deep neural networks. Sustainability.

[10] Pourdaryaei A., Mohammadi M., Mubarak H., Abdellatif A., Karimi M., Gryazina E., Terzija V. (2024). A new framework for electricity price forecasting via multi-head self-attention and cnn-based techniques in the competitive electricity market. Expert Syst. Appl..

[11] Memarzadeh G., Keynia F. (2021). Short-term electricity load and price forecasting by a new optimal lstm-nn based prediction algorithm. Elec. Power Syst. Res..

[12] Li W., Becker D.M. (2021). Day-ahead electricity price prediction applying hybrid models of lstm-based deep learning methods and feature selection algorithms under consideration of market coupling. Energy.

[13] Gao W., Sarlak V., Parsaei M.R., Ferdosi M. (2018). Combination of fuzzy based on a meta-heuristic algorithm to predict electricity price in an electricity markets. Chem. Eng. Res. Des..

[14] Shafi I., Javaid N., Naz A., Amir Y., Ishaq I., Naseem K. (2019). Innovative Mobile and Internet Services in Ubiquitous Computing: Proceedings of the 12th International Conference on Innovative Mobile and Internet Services in Ubiquitous Computing (IMIS-2018).

[15] Syah R., Davarpanah A., Elveny M., Karmaker A., Nasution M., Hossain M. (2021). Forecasting daily electricity price by hybrid model of fractional wavelet transform, feature selection, support vector machine and optimization algorithm. Electronics.

[16] Shafie-Khah M., Moghaddam M.P., Sheikh-El-Eslami M.K. (2011). Price forecasting of day-ahead electricity markets using a hybrid forecast method. Energy Convers. Manag..

[17] Gabrielli P., Wüthrich M., Blume S., Sansavini G. (2022). Data-driven modeling for long-term electricity price forecasting. Energy.

[18] Khan S., Aslam S., Mustafa I., Aslam S. (2021). Short-term electricity price forecasting by employing ensemble empirical mode decomposition and extreme learning machine. Forecasting.

[19] Meng A., Wang P., Zhai G., Zeng C., Chen S., Yang X., Yin H. (2022). Electricity price forecasting with high penetration of renewable energy using attention-based lstm network trained by crisscross optimization. Energy.

[20] Huang C.-J., Shen Y., Chen Y.-H., Chen H.-C. (2021). A novel hybrid deep neural network model for short-term electricity price forecasting. Int. J. Energy Res..

[21] Yang W., Sun S., Hao Y., Wang S. (2022). A novel machine learning-based electricity price forecasting model based on optimal model selection strategy. Energy.

[22] Yang X., Xue M., Guo F., Huang Z., Zhang J. (2017). 2017 Chinese Automation Congress (CAC).

[23] Jiang Y., Huang G., Yang Q., Yan Z., Zhang C. (2019). A novel probabilistic wind speed prediction approach using real time refined variational model decomposition and conditional kernel density estimation. Energy Convers. Manag..

[24] He Y., Zheng Y. (2018). Short-term power load probability density forecasting based on yeo-johnson transformation quantile regression and gaussian kernel function. Energy.

[25] Tang G., Wu Y., Li C., Wong P.K., Xiao Z., An X. (2020). A novel wind speed interval prediction based on error prediction method. IEEE Trans. Industr. Inform..

[26] Koenker R., Hallock K.F. (2001). Quantile regression. J. Econ. Perspect..

[27] Deng Z., Wang B., Guo H., Chai C., Wang Y., Zhu Z. (2020). Unified quantile regression deep neural network with time-cognition for probabilistic residential load forecasting. Complexity.

[28] Shafer G., Vovk V. (2008). A tutorial on conformal prediction. J. Mach. Learn. Res..

[29] Bai S., Kolter J.Z., Koltun V. (2018). An empirical evaluation of generic convolutional and recurrent networks for sequence modeling. arXiv.

[30] Graves A. (2012). Supervised sequence labelling with recurrent neural networks.

[31] Srivastava N., Mansimov E., Salakhudinov R. (2015). International conference on machine learning.

[32] Ikotun A.M., Ezugwu A.E., Abualigah L., Abuhaija B., Heming J. (2023). K-means clustering algorithms: A comprehensive review, variants analysis, and advances in the era of big data. Inf. Sci..

[33] Bagirov A.M., Aliguliyev R.M., Sultanova N. (2023). Finding compact and well-separated clusters: Clustering using silhouette coefficients. Pattern Recognit..

[34] Douglas C.M., Peck E.A., Geoffrey Vining G. (2021).

[35] Pekel E. (2020). Estimation of soil moisture using decision tree regression. Theor. Appl. Climatol..

[36] Sutton C.D. (2005). Classification and regression trees, bagging, and boosting. Handb. Stat..

[37] Breiman L. (2001). Random forests. Mach. Learn..

[38] Zhou J., Zhang L., Zhu L., Zhang W. (2024). A data-driven operating improvement method for the thermal power unit with frequent load changes. Appl. Energy.

[39] Huang N.E., Shen Z., Long S.R., Wu M.C., Shih H.H., Zheng Q., Yen N.-C., Tung C.C., Liu H.H. (1998). The empirical mode decomposition and the hilbert spectrum for nonlinear and non-stationary time series analysis. Proc. R. Soc. Lond. A..

[40] Torres M.E., Colominas M.A., Schlotthauer G., Flandrin P. (2011). 2011 IEEE international conference on acoustics, speech and signal processing (ICASSP).

[41] Dragomiretskiy K., Zosso D. (2014). Variational mode decomposition. IEEE Trans. Signal Process..

[42] Steurer M., Hill R.J., Pfeifer N. (2021). Metrics for evaluating the performance of machine learning based automated valuation models. J. Property Res..

[43] Zhou M., Wang B., Guo S., Watada J. (2021). Multi-objective prediction intervals for wind power forecast based on deep neural networks. Inf. Sci..

