# Supplementary material for: A deep learning-based model for interval prediction of real-time clearing price in electricity market
Source: iScience. 2026 Aug 10;29(8):117075. doi: 10.1016/j.isci.2026.117075 (PMC13486891; doi:10.1016/j.isci.2026.117075)
Supplement: Supplementary file 1 [file mmc1.pdf]

iScience, Volume *29*

Supplemental information

A deep learning-based model for interval prediction of real-time clearing price in electricity market

Jun Yang, Jian Zhou, and Lei Zhu

Supplemental Information

S1 Point prediction performances under regression models

To evaluate the generalizability and effectiveness of the proposed feature construction (FC) and feature selection (FS) methods, four established regression models, Linear Regression (LR), Decision Tree Regression (DT) Bagging Tree Regression (BT), and Random Forest Regression (RF), are employed as benchmarks. These models are used to assess how different feature processing techniques influence predictive performance. Meanwhile, K-means clustering identified two distinct market regimes indicating clear separation between clusters. These regimes, labeled “cluster1” and “cluster2”, reflect notable differences in supply-side composition, particularly in renewable energy output, while electricity demand remains relatively stable. As summarized in Table 1, the average renewable energy output in cluster2 is approximately 5,000 MW, compared to 12,600 MW in cluster1. This divergence in generation mix is directly mirrored in real-time clearing prices, which average 200 CNY/MWh in cluster1 and 501 CNY/MWh in cluster2, underscoring the critical impact of supply structure on market outcomes.

Table S.1: Prediction Performance of Regression Models Across Different Clusters

Model	R-squared	RMSE	MAE	MAPE	SMAPE
LR	0.6447	197.638	109.612	36.94%	27.91%
LR-cluster1	0.5243	125.2654	74.2748	62.87%	36.32%
LR-cluster2	0.5859	220.1302	127.7994	25.88%	22.25%
DT	0.788	152.6561	57.5158	20.83%	15.73%
DT-cluster1	0.6292	110.586	42.9461	38.92%	26.45%
DT-cluster2	0.7546	169.4784	63.4611	12.52%	10.85%
BT	0.8821	113.8509	52.9312	19.22%	13.43%
BT-cluster1	0.7979	81.6372	40.9466	35.10%	21.84%
BT-cluster2	0.8633	126.4808	58.3253	11.78%	9.81%
RF	0.8951	107.3926	49.9533	18.10%	12.57%
RF-cluster1	0.8111	78.9347	39.5289	34.34%	20.92%
RF-cluster2	0.8815	117.7834	54.0695	10.96%	8.99%

The distinct operational characteristics of each cluster significantly influence model performance, as evidenced by the prediction results in Table S.1. A key finding emerges: models exhibit lower absolute errors (RMSE, MAE) in Cluster 1, while demonstrating superior performance in relative error metrics (MAPE, SMAPE) in Cluster 2. This seeming discrepancy stems from fundamental differences in price distributions: Cluster 1’s lower absolute price levels naturally lead to smaller absolute errors but can amplify percentage-based errors. In contrast, the higher-price environment of Cluster 2 results in larger absolute deviations but smaller relative errors when scaled against the prevailing price level. This analysis highlights how the distributional properties of the target variable directly shape the interpretation of predictive accuracy. Moreover,

it underscores the growing necessity for robust forecasting frameworks, especially as rising renewable energy integration introduces heightened volatility and complexity into electricity market dynamics.

Table S.2: Prediction Performance of Regression Models Under Different Feature Preprocessing Methods

Model	R-squared	RMSE	MAE	MAPE	SMAPE
LR	0.6447	197.6380	109.6120	36.94%	27.91%
LR-FS	0.6440	197.8266	109.4413	36.78%	27.82%
FC-LR	0.9282	88.2905	35.9438	13.49%	10.60%
FC-LR-FS	0.9257	89.8107	35.1719	13.09%	10.22%
DT	0.7880	152.6561	57.5158	20.83%	15.73%
DT-FS	0.7874	152.8999	57.3549	20.34%	15.39%
FC-DT	0.8704	118.5865	43.9364	16.02%	12.68%
FC-DT-FS	0.8625	122.1395	45.5230	16.22%	13.05%
BT	0.8821	113.8509	52.9312	19.22%	13.43%
BT-FS	0.8770	116.2971	54.0492	19.28%	13.60%
FC-BT	0.9252	90.0791	35.7985	13.66%	9.94%
FC-BT-FS	0.9222	91.9088	36.5705	13.57%	9.99%
RF	0.8951	107.3926	49.9533	18.10%	12.57%
RF-FS	0.8933	108.3235	50.7931	18.32%	12.73%
FC-RF	0.9306	86.7647	33.9934	12.87%	9.32%
FC-RF-FS	0.9290	87.8062	34.8305	13.09%	9.49%

Notes: The number of input features for the base model is 21. This is reduced to 16 with FS, expanded to 49 with FC, and optimized to 15 with the FC-FS combination.

To evaluate the impact of feature preprocessing on model performance, four distinct strategies were implemented: a base model without preprocessing, feature selection (FS) alone, feature construction (FC) alone, and their combined application (FC–FS). As summarized in Table S.2, the predictive performance of four regression models under these strategies reveals clear differences in effectiveness. Greedy-based feature selection consistently reduced model complexity with only marginal performance loss, as evidenced by comparisons such as BT versus BT–FS and FC–BT versus FC–BT–FS, where FS maintained predictive accuracy across all metrics while using fewer features and thus improving computational efficiency and model parsimony. In contrast, time-shift-based feature construction significantly enhanced predictive performance, with models incorporating FC (e.g., FC–BT and FC–RF) outperforming their counterparts across all evaluation metrics. This improvement underscores the ability of FC to capture short-term temporal patterns in real-time electricity prices, a dependency not inherently modeled by standard regression techniques, as further supported by the trends illustrated in Figure 2c. Overall, the combined FC–FS approach retained most of the performance gains from feature construction while benefiting from the dimensionality reduction of feature selection, offering a balanced and practical solution for large-scale electricity market applications.

S2 Detailed algorithmic parameters and piecewise interval integration

To precisely quantify the uncertainty inherent in electricity price forecasting and to operationalize the proposed three-step interval construction framework, specific algorithmic parameters were instantiated based on the regulatory constraints and empirical distribution of the data. As illustrated in Figure 2a, the market exhibits a highly concentrated normal operating regime alongside heavily skewed, asymmetric extreme prices bounded by regulatory caps. In the first step, i.e., similar point matching, historical similarity is evaluated using a cosine similarity threshold of no less than 0.99, with the candidate pool strictly restricted to prior timestamps to prevent data leakage, and the maximum number of retrieved candidates set to $n = 1000$.

Second, for statistical characterization, the empirical prices of the matched candidates are discretized into $K = 10$ equal-frequency quantile bins; to ensure robustness against outliers, low-frequency bins with empirical probability below 11% are filtered out, thereby establishing stable empirical boundary intervals $[\mathrm{Bin}_{i-1}, \mathrm{Bin}_i]$.

Finally, during interval integration, the adaptive prediction interval $[L_t, U_t]$ is generated via a piecewise function anchored to the LSTM point prediction $\hat{y}_t$. This approach translates the market's regulatory constraints and historical empirical boundaries directly into executable rules without relying on arbitrary statistical assumptions. Specifically, when the point prediction falls within the high-density stable regime ($200 \leq \hat{y}_t \leq 600$), where market volatility is fundamentally minimal, a tight proportional buffer is applied: $[0.9\hat{y}_t, 1.1\hat{y}_t]$. In extreme high-price scenarios ($\hat{y}_t \geq 1300$), the interval is strictly clamped to $[1300, 1500]$, explicitly enforcing the regulatory ceiling. For standard market fluctuations where $\hat{y}_t$ lies within a valid historical empirical bin $[\mathrm{Bin}_{i-1}, \mathrm{Bin}_i]$, a localized dynamic buffer is used: $L_t = \min(\mathrm{Bin}_{i-1}, \hat{y}_t - 20)$ and $U_t = \min(\mathrm{Bin}_i, \hat{y}_t + 20)$, ensuring a responsive margin of ± 20 while remaining bounded by actual historical edges to avoid overestimating uncertainty. For out-of-distribution predictions, tailored asymmetric logic is deployed: if $\hat{y}_t < \mathrm{Bin}_0$, the bounds are conservatively scaled downward to $[0.5\mathrm{Bin}_0, 0.5(\mathrm{Bin}_0 + \hat{y}_t)]$ to establish a safe floor; conversely, if $\hat{y}_t > \mathrm{Bin}_{\max}$, the interval is solidly bounded between the maximum empirical edge and the 1500 CNY/MWh regulatory cap.

S3 Configuration of comparative baselines

To rigorously evaluate the effectiveness of this proposed nonparametric empirical model, comparative experiments were conducted against three distinct probabilistic forecasting paradigms. The first is a naive error-based reference interval, constructed by scaling the point prediction by factors of 1.1 and 0.9, heuristically reflecting the approximately 10% MAPE of the underlying LSTM.

The second and third baselines belong to the Quantile Regression (QR) paradigm, representing end-to-end parametric probabilistic approaches. To establish a standard parametric baseline, the underlying LSTM architecture was modified with a multi-quantile output head (LSTM-QR). Furthermore, to rigorously benchmark against current state-of-the-art (SOTA) methodologies, a Temporal Convolutional Network combined

with Quantile Regression (TCN-QR) was implemented. And both QR networks were optimized directly via the pinball loss function. To explicitly investigate the inherent trade-off between coverage probability and interval sharpness in parametric models, we systematically trained both the LSTM-QR and TCN-QR models across a sweeping spectrum of nominal theoretical coverages: 80%, 82%, 84%, 86%, 88%, and 90% (by predicting the quantile pairs [0.10, 0.90], [0.09, 0.91], [0.08, 0.92], [0.07, 0.93], [0.06, 0.94], and [0.05, 0.95], respectively). Evaluating this spectrum allows for an objective benchmarking against our proposed model. Crucially, these parametric QR models were applied exclusively to the undecomposed feature sets, as applying quantile regression to decomposed sub-series is mathematically problematic. Since the variance of a joint distribution is not strictly additive, decomposing the signal distorts its original joint probability distribution and extreme tail properties.

The four baseline is Conformal Prediction (CP). Unlike QR, CP is a rigorous statistical post-processing framework rather than an end-to-end model. It was applied to the point prediction of all evaluated models to provide distribution-free uncertainty bounds. Adhering to the aforementioned comparative rationale, the significance level of the CP algorithm was not strictly fixed, but dynamically calibrated to precisely match the empirical PICP of the proposed interval generator. This dynamic alignment guarantees that the subsequent comparison of MIW and MPICD remains purely focused on interval sharpness and calibration, unbiased by coverage discrepancies.
